# Over-Representation of Torque Teno Mini Virus 9 in a Subgroup of Patients with Myalgic Encephalomyelitis/Chronic Fatigue Syndrome: A Pilot Study

**DOI:** 10.3390/pathogens13090751

**Published:** 2024-09-01

**Authors:** Karen Giménez-Orenga, Eva Martín-Martínez, Elisa Oltra

**Affiliations:** 1 Escuela de Doctorado, Universidad Católica de Valencia San Vicente Mártir, 46001 Valencia, Spain; karen.gimenez@mail.ucv.es; 2 National Health Service, Manises Hospital, 46940 Valencia, Spain; evamariamartinmartinez@gmail.com; 3 Department of Pathology, School of Medicine and Health Sciences, Universidad Católica de Valencia San Vicente Mártir, 46001 Valencia, Spain

**Keywords:** ME/CFS, FM, TTMV, Anellovirus, Betatorquetevirus family, microarray

## Abstract

Myalgic encephalomyelitis/chronic fatigue syndrome (ME/CFS) is a chronic disorder classified by the WHO as postviral fatigue syndrome (ICD-11 8E49 code). Diagnosing ME/CFS, often overlapping with fibromyalgia (FM), is challenging due to nonspecific symptoms and lack of biomarkers. The etiology of ME/CFS and FM is poorly understood, but evidence suggests viral infections play a critical role. This study employs microarray technology to quantitate viral RNA levels in immune cells from ME/CFS, FM, or co-diagnosed cases, and healthy controls. The results show significant overexpression of the Torque Teno Mini Virus 9 (TTMV9) in a subgroup of ME/CFS patients which correlate with abnormal HERV and immunological profiles. Increased levels of TTMV9 transcripts accurately discriminate this subgroup of ME/CFS patients from the other study groups, showcasing its potential as biomarker for patient stratification and the need for further research into its role in the disease. Validation of the findings seems granted in extended cohorts by continuation studies.

## 1. Introduction

Myalgic encephalomyelitis/chronic fatigue syndrome (ME/CFS) is a chronic complex disorder classified by the WHO with the ICD-11 8E49 code as a postviral fatigue syndrome [1]. It is characterized by debilitating fatigue that is not alleviated by rest, generalized pain, sleep disturbances, cognitive impairments, and a variety of other symptoms that can severely impact daily functioning [2]. Diagnosis of ME/CFS is based on the clinical assessment of nonspecific symptoms and frequently overlaps with fibromyalgia (FM) (ICD-11 MG30.0 for chronic primary pain) [3] (Carruthers et al. 2011a; Carruthers et al. 2011b). FM is a chronic disorder characterized by a low pain threshold, stiffness and tenderness in the muscles of the neck, usually accompanied by fatigue, memory loss, and sleep disturbances [4,5]. Their frequent co-diagnosis, and the lack of biomarkers for either condition, have prompted the exploration of both common and unique molecular factors [6,7,8,9] that may explain their frequent joint appearance and aid in differential diagnosis.

The absence of reliable biomarkers is partly due to the limited understanding of the disease, as the precise etiology of ME/CFS and FM remain elusive. Nevertheless, a growing body of evidence suggests that viral infections may play a pivotal role in the onset and progression of the disease [10,11,12,13,14]. Active viral infections may cause immunological disturbances like autoimmunity or immunological dysfunction [15,16,17] and trigger dramatic changes in the epigenetic landscape [18], leading to the viral reactivation of exogenous [19,20,21] or endogenous retroviruses [22]. This may contribute to the chronic immune activation and dysregulation observed in ME/CFS patients [23], as well as the persistence and exacerbation of symptoms.

Up to date, no single infectious agent has been associated with ME/CFS, as reviewed by Rasa et al. [14]. Conflicting research results and experimental limitations like heterogeneous ME/CFS cohorts, a high prevalence of persistent viral infection in the general population, and different methodological approaches, among other factors, may have hindered this finding. In a previous work, we identified the reactivation of human endogenous retroviral sequences in a subgroup of ME/CFS patients whose expression correlated with an altered immunological profile, with the results available as a preprint [9]. Those features allowed for the perfect discrimination of that ME/CFS subgroup, suggesting alterations in patients’ epigenetic landscape.

The aim of this study was to identify candidate exogenous viral RNA sequences that may associate with the HERV activation profile and correlated immunological disturbances observed in the described subset of ME/CFS patients. To address this question, we performed a comprehensive analysis through microarray technology of viral RNA sequences present in the exact same RNA samples analyzed in our previous study, focusing on increased viral load in the subgroup of ME/CFS patients exhibiting altered HERV profiles as compared to FM, co-diagnosed cases, and healthy controls. Our findings revealed the significant overexpression of the Torque Teno Mini Virus 9 (TTMV9) in the immune cells of this subgroup of ME/CFS patients compared to the other study groups. The identification of TTMV9 and its association with a particular subset of ME/CFS cases provides new insights into the viral mechanisms that may underlie this complex disorder and opens avenues for further research into, diagnostic markers, targeted treatments, and prevention programs.

## 2. Materials and Methods

### 2.1. Participant Recruitment

This cross-sectional observational study was approved by the Public Health Research Ethics Committee DGSP-CSISP of Valencia, num. 20190301/12, Valencia, Spain. This study included a total of 34 female patients from local patient associations (National Biobank Registry Ref. 0006024) who were clinically diagnosed with ME/CFS (*n* = 8) according to the Canadian (Carruthers et al. 2011a) and International Consensus (Carruthers et al. 2011b) criteria, with FM (*n* = 10) according to the 1990 [4] and 2011 [5] American College of Rheumatology (ACR) criteria for FM, or both, ME/CFS and FM (*n* = 16), hereafter referred to as the co-diagnosed group. A healthy control group of individuals population-matched for age and BMI consisting of 9 female donors was also included in this study (National Biobank Registry Ref. 0006034). Patients with health problems other than FM and ME/CFS were excluded. Individuals with any similar or related pathology, including a medical history of chronic pain and/or fatigue, or serious health complications, were excluded from control group, as well as medicated healthy controls. Written informed consent was obtained from all study participants.

### 2.2. Blood Sample Collection, Processing and Storage

After a 12 h overnight fasting and medication withdrawal, whole blood was extracted from participants following the protocol described in Ref. [9]. In brief, up to 10 mL of whole blood was collected via venipuncture in K2EDTA tubes (Becton Dickinson, Franklin Lakes, NJ, USA) and processed within 2 h. Peripheral blood mononuclear cells (PBMCs) were isolated by dilution at 1:1 (*v*/*v*) ratio in phosphate-buffered saline solution (PBS) with layering on top of 1 volume of Ficoll-Paque Premium (GE Healthcare, Chicago, IL, USA) and separation by density centrifugation at 500× *g* for 30 min (20 °C, brakes off). The PBMC layer was washed with PBS, contaminant erythrocytes were removed, and cells adjusted to a final concentration of 10^7^ cells/mL in freezing medium (90% FBS, 10% DMSO), aliquoted, and deeply frozen in liquid nitrogen until use.

### 2.3. RNA Extraction, Processing and Analysis by High-Density Microarray

Total RNA from PBMCs was extracted using RNeasy Mini Kit (Qiagen, Hilden, Germany) according to the manufacturer’s instructions. RNA quality was assessed using Agilent TapeStation 4200 (Agilent Technologies, Santa Clara, CA, USA). All RNA samples had an RNA Integrity number (RIN) above seven. To evaluate the presence of foreign viral RNA sequences, cDNA was synthesized and amplified from 45 ng of RNA using the Ovation Pico WTA System V2 kit (Tecan, Männedorf, Switzerland) according to the manufacturer’s instructions. The resulting amplified ssDNA was purified using the QIAquick purification kit (Qiagen, Hilden, Germany). Total DNA concentration was measured with a NanoDrop 2000 spectrophotometer (Thermo Scientific, Waltham, MA, USA) and quality was assessed on the Bioanalyzer 2100 (Agilent Technologies, Santa Clara, CA, USA). Purified ssDNA was fragmented, biotin-labeled, hybridized, and read according to the protocol described in detail in Ref. [9] by Sampled (Piscataway, NJ, USA). The custom high-density HERV-V3 microarrays [24] were used to detect the presence of 289 exogenous infectious virus, including the following: (i) dsDNA viruses (103): adenovirus, herpesvirus, polyomavirus, papillomavirus; (ii) RT viruses (82): ALV, BFV, ELL, FFV, FLV, FIV, MLV, HIV, HTVL, SIV, KoRV, etc.; (iii) ssDNA viruses (31): Torque virus; and (iv) ssRNA viruses (75): hepatitis C, dengue, hantaviruses, influenza, coronavirus (full list is provided with the MTA to users).

### 2.4. Bioinformatic Analysis

All bioinformatic analyses were performed with RStudio software version 4.2.1. Microarray CEL files were processed and analyzed using R oligo package [25]. Data were normalized, adjusted for background noise, and summarized using the robust multi-array (RMA) algorithm. Differential expression (DE) analysis was performed using limma R package [26], considering differentially expressed those probes with a “Benjamin-Hochberg” (BH) adjusted *p* value < 0.05 and an absolute log2 fold-change > 1. Plots were represented with ggplot2 R package [27]. Correlation analyses were performed with the package Hmisc v5.1. The ME/CFS diagnosis variable was binarized in order to be compared with continuous variables. Functional analysis by gene ontology of biological processes was performed with ShinyGO 8.0 [28]. Digital cytometry analysis was performed with CIBERSORTx online tool (https://cibersortx.stanford.edu, accessed on 15 May 2024), as previously described [29], on normalized non-logarithmic gene expression data for all samples. The default LM22 leukocyte gene signature matrix [30] was used as a reference. Response operating characteristic (ROC) analysis with area under curve (AUC) were used to assess the discriminating capacity of the variable and were performed with the R package pROC version 1.18.5 [31].

### 2.5. Statistical Analysis

Continuous variables were presented as mean ± standard deviation. All statistical analyses were performed in R v4.2.1. Data distributions were tested for normality. Normally distributed data were tested using two-tailed unpaired Student’s *t*-tests; non-normal data were analyzed with non-parametric statistical test, as detailed.

## 3. Results

### 3.1. Clinical Characteristics of Participants

This study included 43 pre-pandemic female participants aged 42 to 58 years, with cases: *n* = 8 ME/CFS, *n* = 10 FM, *n* = 16 ME/CFS + FM (co-diagnosed), and *n* = 9 matched healthy controls. According to a previous study on the same cohort, ME/CFS cases were stratified into two subgroups based on gene and human endogenous retrovirus (HERV) expression, the ME/CFS subgroup 1 (*n* = 3) and the ME/CFS subgroup 2 (*n* = 5) [9]. Patient symptoms were assessed through the Fibromyalgia Impact Questionnaire (FIQ) [32], Multi Fatigue Inventory (MFI) for general fatigue [33], and Short-Form-36 Health Survey (SF-36) [34] for quality of life. Itemized questionnaire scores can be accessed in the reference study published by our group [9]. Significant differences were only found between the ME/CFS subgroup 2 and the co-diagnosed groups for MFI general fatigue (19 ± 1.7 vs. 13.6 ± 3.2, respectively, *p* = 0.035) (Figure S1), as previously reported [9]. Nonetheless, a tendency for lower scores with *p <* 0.1 could be observed for other items of the MFI questionnaire in ME/CFS subgroup 2, suggesting a worse health status for the patients included in the group.

### 3.2. Increased Levels of TTMV9 in PBMCs of a Subgroup of ME/CFS Cases

**Figure 1:**
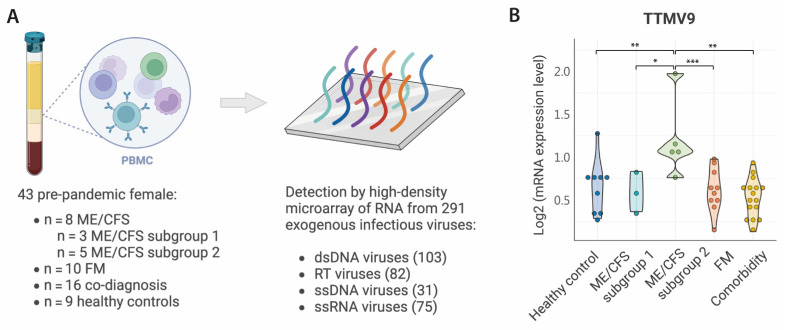
Analysis by high-density microarray of RNA viral sequences in PBMC from patients and controls. (**A**) Schematic description of the study design. RNA was extracted from isolated PBMCs of participants and further processed for its analysis by HERV-V3 high density microarrays detecting RNA viral sequences from up to 289 viruses. (**B**) Violin plots summarize the distribution and expression levels of TTMV9 in each study group. Statistical tests: unpaired two-sample Wilcoxon Test with Benjamini–Hochberg *p*-value correction (* *p* < 0.05, ** *p* < 0.01, *** *p* < 0.001).

To identify exogenous viral RNA potentially causing the activation of HERVs in a subgroup of ME/CFS cases, we analyzed using high-density microarray the expression levels in PBMC of up to 289 viruses belonging to the following eleven families (Figure 1A): (i) Retro-transcribing viruses: *Hepadnaviridae, Retroviridae*; (ii) ssRNA viruses: ssRNA-positive-strand viruses with no DNA stage, *Coronaviridae*; (iii) dsDNA viruses: *Mastadenovirus, Chordopoxvirinae, Herpesviridae, Alphapapillomavirus, Betapapillomavirus, Gammapapillomavirus*; and (iv) ssDNA viruses: *Anelloviridae* (Table S1).

The overall analysis of viral RNA by family did not reveal any significant changes associated with ME/CFS subgroup 2 compared to the others. Differences were only detected for the *Coronaviridae, Gammapapillomavirus*, and *Retroviridae* families, and only between FM and ME/CFS subgroup 2 (Figure S2). Individual analysis of the viruses from each of these families revealed that only for some viruses were these differences confirmed (Figures S3–S5). As we aimed to identify viral RNA sequences discriminating the ME/CFS subgroup 2 from all the other study groups, we independently compared the expression of each of the 289 viral sequences included in the microarray. Surprisingly, only one virus, the Torque Teno Mini Virus 9 (TTMV9) from the family *Anelloviridae,* was specific for the subgroup 2 of ME/CFS patients, with levels above all the other study groups (Figure 1B). Interestingly, TTMV9 was the only member of the *Anelloviridae* family with increased RNA levels in this disease group (Figure S6) and would have been overlooked if the analysis had been conducted only at the family level.

### 3.3. TTMV9 Levels Correlate with DE HERVs and Genes

**Figure 2:**
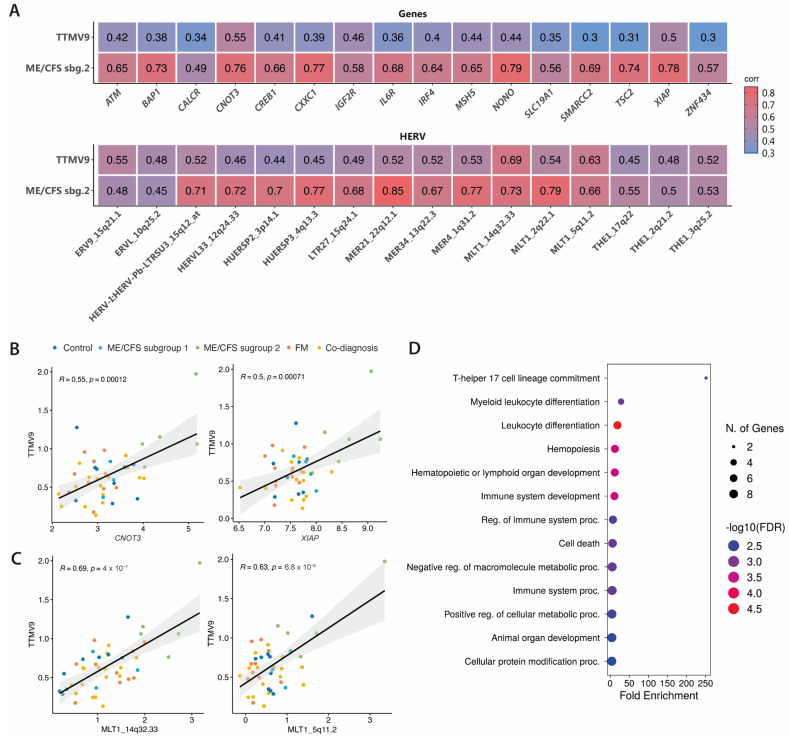
Levels of TTMV9 correlate with activation of HERVs and differential expression of immune-related genes. (**A**) Heatmap representation of Pearson correlations between TTMV9 RNA levels, activated HERVs, and DE genes in the ME/CFS patient subgroup 2. The heatmap also shows correlations between HERV and gene profiles with ME/CFS diagnosis. Boxes show Pearson correlation values of only statistically significant correlations (*p* < 0.05). A value of 1 (red) and 0 (blue) quantify strongest and weakest positive correlations, respectively. (**B**) Top correlated genes with TTMV9 expression. (**C**) Top correlated HERVs with TTMV9 expression. (**D**) Gene ontology analysis by biological process of genes significantly correlated with TTMV9 RNA levels.

To understand if the increased viral RNA of TTMV9 is associated with the molecular fingerprint previously identified in the same subgroup of ME/CFS patients [9], we searched for potential correlations between the levels of TTMV9 and the associated HERV and gene signatures in all study groups [9] (Figure 2). Moderate correlations with TTMV9 levels were observed for the top DE genes, especially for *CNOT3* (*R* = 0.55, *p* = 0.00012) and *XIAP* (X-linked inhibitor of apoptosis protein) (*R* = 0.5, *p* = 0.00071) (Figure 2A,B). Interestingly, the genes showing the highest correlation with TTMV9 levels were also strongly correlated with ME/CFS diagnosis for subgroup 2 (as previously published in our preprint [9]) (*R* = 0.76, *p* < 0.0001 and *R* = 0.78, *p* < 0.0001, respectively; Figure 2A), indicating the potential involvement of TTMV9 levels with disease and gene expression in this patient subgroup. Similarly, HERV levels, highly correlating with ME/CFS diagnosis for subgroup 2 [9], showed moderate to strong associations with TTMV9 expression, e.g., MLT1_14q32.33 (*R* = 0.69, *p* = 0.00007), located within the lncRNA *LINC02298*, and MLT1_5q11.2 (*R* = 0.63, *p* < 0.0001), 25 kbp away from the gene *COX6C* (Figure 2A,C). Functional analysis of the genes significantly correlated with TTMV9 levels revealed enrichment for the pathway involved in the commitment of CD4 T cells towards T-helper 17 cells and other leukocyte differentiation events (Figure 2D).

### 3.4. TTMV9 Levels Correlate with Plasma Cell and Gamma Delta T Cell Population Levels and Discriminate ME/CFS Subgroup 2 from All Other Study Groups

**Figure 3:**
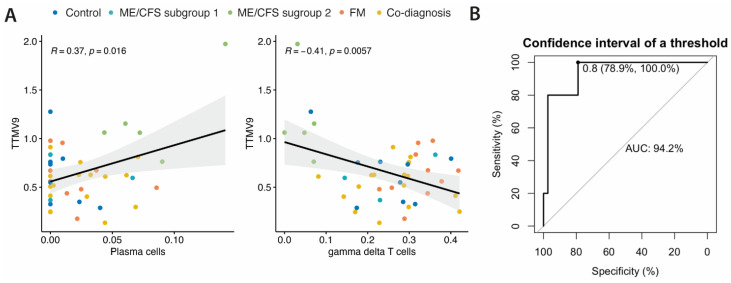
(**A**) Pearson correlation between TTMV9 levels and immune cell ratios. (**B**) Receiver operating characteristics (ROC) analysis of TTMV expression levels measured by high density microarray in ME/CFS subgroup 2 (*n* = 5) vs. ME/CFS subgroup 1 (*n* = 3), FM (*n* = 10), comorbidity (*n* = 16), and healthy controls (*n* = 9). The area under the curve (AUC) and the best threshold value with the corresponding specificity and sensitivity appear indicated.

Given the correlation of TTMV9 levels with genes involved in T-cell differentiation pathways, and the previously reported association between the ME/CFS subgroup 2- HERV fingerprint and certain immune cell populations, as described in the preprint [9], we sought to investigate the relationship between TTMV9 levels and immune cell proportions. To this end, we studied potential correlations of the frequencies of each cell type with TTMV9 RNA levels, using CIBERSORTx online tool (https://cibersortx.stanford.edu, accessed on 15/05/2024), as previously described [29]. Interestingly enough, significant correlations were only found for plasma cells (*R* = 0.37, *p* = 0.016) and γδ T cells (*R* = −0.41, *p* = 0.0057) (Figure 3A), coinciding with that already reported for HERV fingerprints in this ME/CFS subgroup [9], suggesting selective effects on these cell types by the virus.

Lastly, to assess the discriminating power of TTMV9 RNA levels and its potential as a biomarker of the ME/CFS subgroup 2, we performed receiver operating characteristics (ROC) analysis. The area under the curve (AUC) was above 0.9, reaching 100% sensitivity and 78.9% specificity, indicative of good capacity to discriminate this subgroup of ME/CFS cases not only from healthy subjects, but also from FM and co-diagnosed cases.

## 4. Discussion

The use of high-density microarrays containing probe sets with the capacity to detect active transcription for 289 exogenous infectious viruses has allowed us to rule out differential expression of EBV, herpes, enterovirus, or others in our ME/CFS cohort. This is a relevant aspect since many have been formerly detected in other cohorts [35]. It has also allowed us to study the correlation of differential active infection of the TTMV virus with HERV and immune gene profiles. The main limitations of this study are the reduced number of cases per study group, and the research of viral sequences not detectable by our microarrays, particularly some previously reported for ME/CFS, including CMV, Borna disease virus, or Parvovirus 19 [35], or the effects of coinfections with bacteria or other microorganisms.

Torque Teno Mini Viruses (TTMV) are non-enveloped and circular ssDNA viruses belonging to the genus *Betatorquevirus* from the family *Anelloviridae* [36]. There are multiple species of TTMV, among which we can find TTMV9 [36]. Anelloviruses (AV) are a major component of the human virome ubiquitously present in the human population across different tissues in the human body [37]. Interestingly, the “anellome” of each individual is unique, being comprised of multiple AV genera and species in different ratios [38]. Despite being acquired at an early stage of life, no pathogenic role has been attributed to their presence. However, high AV titers are considered an indicator of weak immune competence [39] and may potentially reflect the immunocompromised status of some ME/CFS patients. Furthermore, AV load has been shown to be altered in different pathologies, including diverse cancer types or pathologies of the immune system, diabetes, intestinal bowel disease, or in persistent infections such as hepatitis or AIDS [40,41]. Perhaps ME/CFS, or at least a subset of cases, similarly to our subgroup 2, will be added to this list of chronic pathologies once these findings are validated at the epidemiological level. In support of the compromised immunocompetence of these patients, the few statistically significant differences found in this study also involved the increased presence in ME/CFS group 2 (e.g., human papilloma virus 5).

The study of TTMV has been hampered by the lack of in vitro culture systems. However, recent advances on the structure of Anellovirus particles indicates that the strategy they use for immune evasion is to expose highly diverse epitopes acting as immune decoys that elicit weak immune responses, while hiding conserved regions, which permits multiple strains to coexist in an individual [42]. This brings up the question as to what may be different in the TTMV9 from other TTMV species to become prominently active in this subgroup of ME/CFS patients to cause or sustain disease. A possibility may come from inclined instability of the conserved domains, perhaps leading to autoimmunity through molecular mimicry events, as described for lupus and the HRES-1 endogenous retrovirus encoded autoantigen [43,44]. Continuation studies pursuing serological reactivity titrations of these patients’ blood against MMTV9, as well as against the human proteome, should clarify this possibility.

The finding of increased numbers of plasma cells with increased levels of TTMV9 RNA could just reflect the response to infection, perhaps at a chronic stage in these patients. However, in addition to humoral immunity, other roles in hematopoiesis and neuro-inflammation have been attributed to this cell type offering other potential interventions [45]. On another end, reduced ϒδT cell numbers may indicate increased susceptibility to infections in these subjects.

Through the identified correlations of increased TTMV9 levels and DE genes, and the association of the latter with patient symptoms, as previously published [9], it can be inferred that increased levels of TTMV9, may directly or indirectly relate with increased “General fatigue” scores, as measured by the MFI instrument, and with worsening of “Role Emotional” and “Mental health” domains of the SF-36 questionnaire. In addition, the finding of this viral strain in the blood and cerebrospinal fluid of a pediatric case of encephalitis [46] seems to set a pathogenic spectrum for the virus involving the CNS. Although the relationship between TTMV9 and *CNOT3* or *XIAP* levels could be cause, consequence, or even coincidence (unlikely according to statistic parameters), it should be mentioned that altered levels of CNOT3 and other CNOT members or XIAP triggered by infections have been described [47,48]. In addition to potential biomarker value, the understanding of the mechanisms leading to increased XIAP in ME/CFS, either due by activation of TTMV or not, suggests the potential benefit of embelin, a plant-derived benzoquinone with anti-oxidant and anti-inflammatory properties targeting XIAP [49]. However, the validation of our findings in extended cohorts of patients seem required before exploring this possibility.

Lastly, the potential effects of TTMV9 through HERV elements are more difficult to envision as their functions are mainly unknown. However, molecular effects through the associated competitive endogenous (ceRNA) of miR-140-3p-*GPRIN1* axis of *LINC02298* [50], or through cytochrome c oxidase subunit 6C (COX6C) function, as found in other neurological diseases [51], may still be possible.

## 5. Conclusions

In summary, this study identifies the overexpression of TTMV9 viral RNA correlating with activation of particular DE genes and HERV elements, coinciding with increased plasma and decreased ϒδT cell numbers, in a subgroup of ME/CFS patients not fulfilling FM clinical criteria. Patient subgroup phenotype is characterized by the worsening of symptoms with increased TTMV9 expression, mainly involving fatigue and emotional–mental health status. The mechanism behind TTMV9 correlation with patient symptoms, seems to deserve further exploration for the advancement of proper patient classification and the development of personalized medicine programs to effectively treat ME/CFS.

## Data Availability

The original contributions presented in this study are included in the article/supplementary materials, or previous publications, as indicated. Further inquiries can be directed to the corresponding author/s.

## Supplementary Materials

The following supporting information can be downloaded at: https://www.mdpi.com/article/10.3390/pathogens13090751/s1, Table S1: differential expression analysis of viral sequences analyzed by high-density microarray HERV-V3, Figure S1: patient health status assessment through questionnaires, Figure S2: virus families represented in the HERV-V3 microarray; Figure S3: detailed analysis of viruses belonging to the *Coronaviridae* family; Figure S4: detailed analysis of viruses belonging to the *Gammapapillomavirus* family; Figure S5: detailed analysis of viruses belonging to the *Retroviridae* family; Figure S6: detailed analysis of viruses belonging to the *Anelloviridae* family.
