# Supplementary material for: Over-Representation of Torque Teno Mini Virus 9 in a Subgroup of Patients with Myalgic Encephalomyelitis/Chronic Fatigue Syndrome: A Pilot Study
Source: Pathogens. 2024 Sep 1;13(9):751. doi: 10.3390/pathogens13090751 (PMC11435283; doi:10.3390/pathogens13090751)
Supplement: Supplementary file 1 [file pathogens-13-00751-s001.zip › Figures S1-S6.pdf]

pathogens

MDPI

Supplementary materials

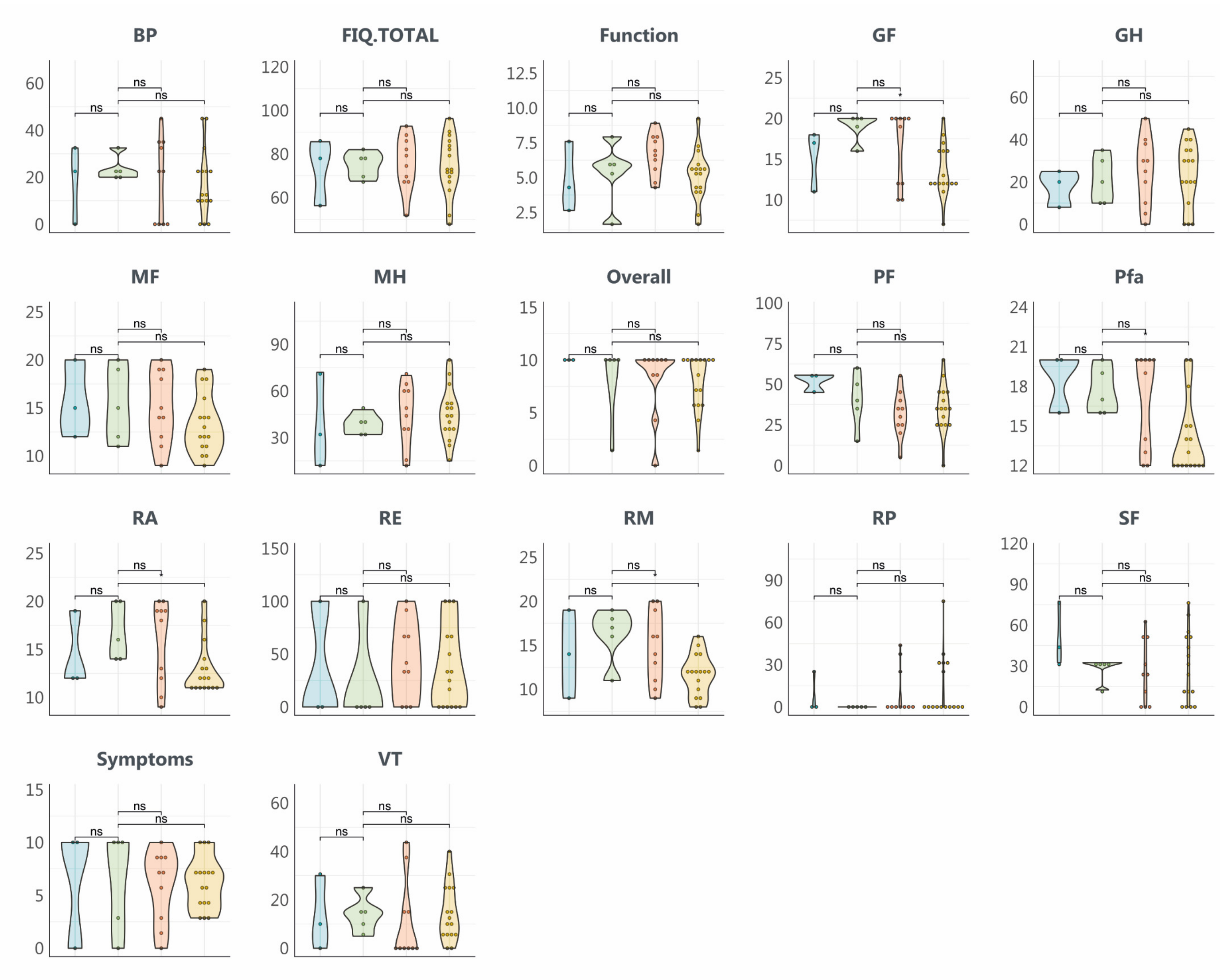

Figure S1. Patient Health Status assessment through questionnaires. FIQ subdomains (Function, Overall and Symptoms) or total FIQ; for MFI subdomains (GF: general fatigue, Pfa: physical fatigue, RA: reduced activity, RM: reduced motivation, and MF: mental fatigue), and for SF-36 subdomains (PF: physical functioning, RP: role physical, BP: bodily pain, GH: general health, VT: vitality, SF: social functioning, RE: role emotional, and MH: mental health). (*p<0.05, ns: non-significant).

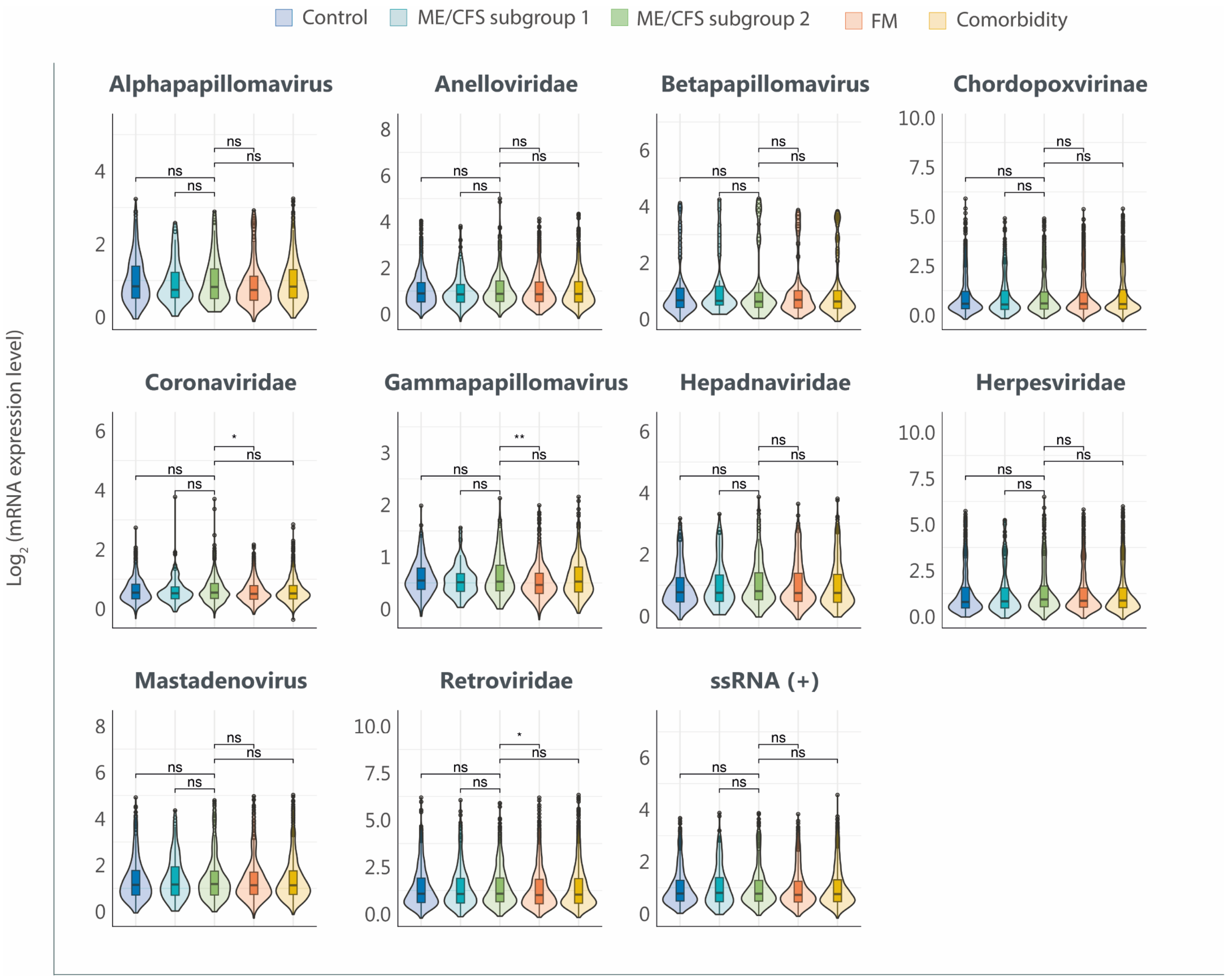

Figure S2. Virus families represented in the HERV-V3 microarray. Plots summarize the distribution and expression levels of all probesets of viruses belonging to each family in each study group. Statistical tests: unpaired two-sample Wilcoxon Test with Benjamin-Hochberg p-value correction. (*p<0.05, **p<0.01, ns: non-significant).

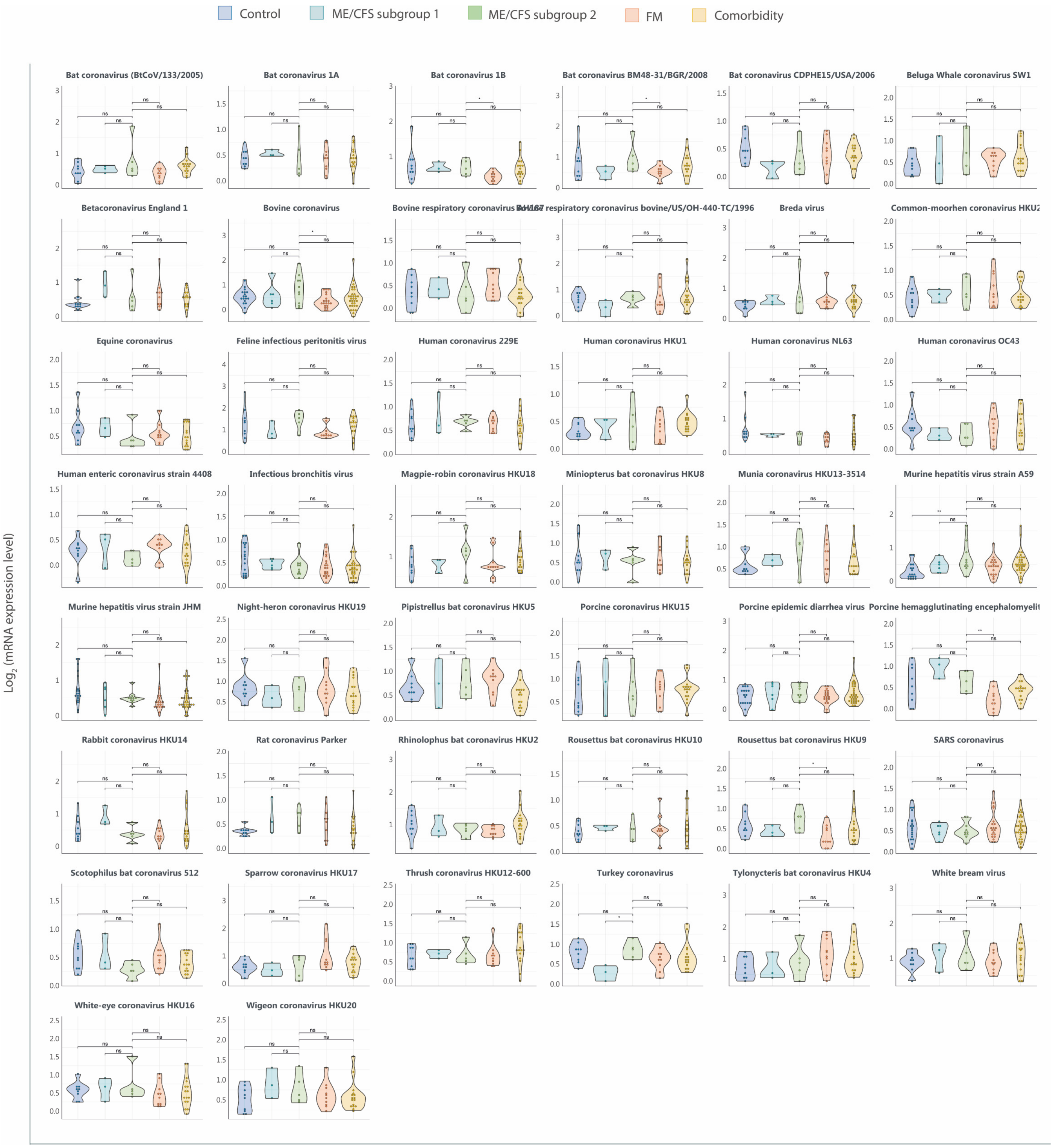

Figure S3. Detailed analysis of viruses belonging to the *Coronaviridae* family. Plots summarize the distribution and expression levels of all probesets of the corresponding virus in each study group. Statistical tests: unpaired two-sample Wilcoxon Test with Benjamin-Hochberg p-value correction. (*p<0.05, **p<0.01, ns: non-significant).

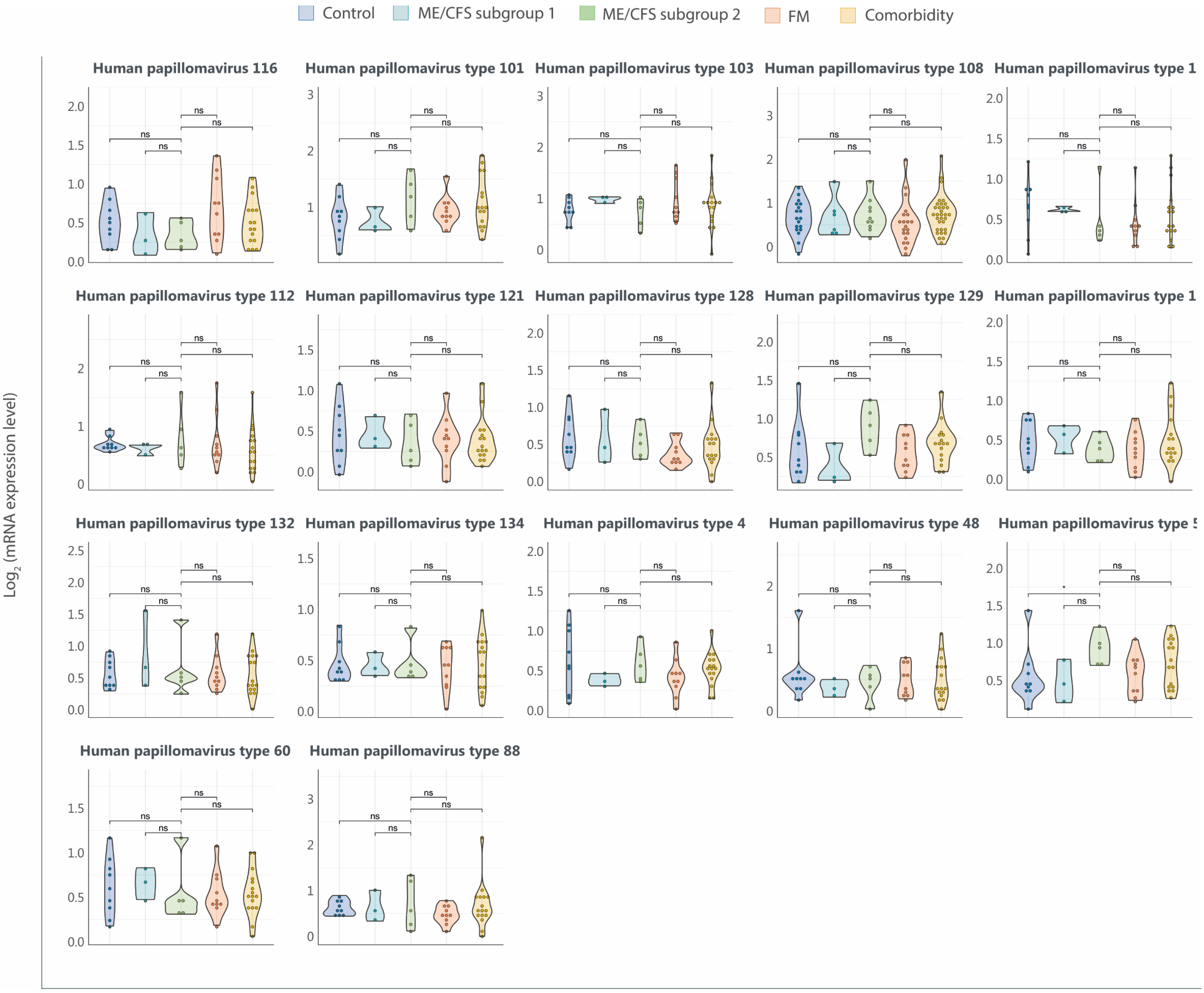

Figure S4. Detailed analysis of viruses belonging to the *Gammapapillomavirus* family. Plots summarize the distribution and expression levels of all probesets of the corresponding virus in each study group. Statistical tests: unpaired two-sample Wilcoxon Test with Benjamin-Hochberg p-value correction. (*p<0.05, **p<0.01, ns: non-significant).

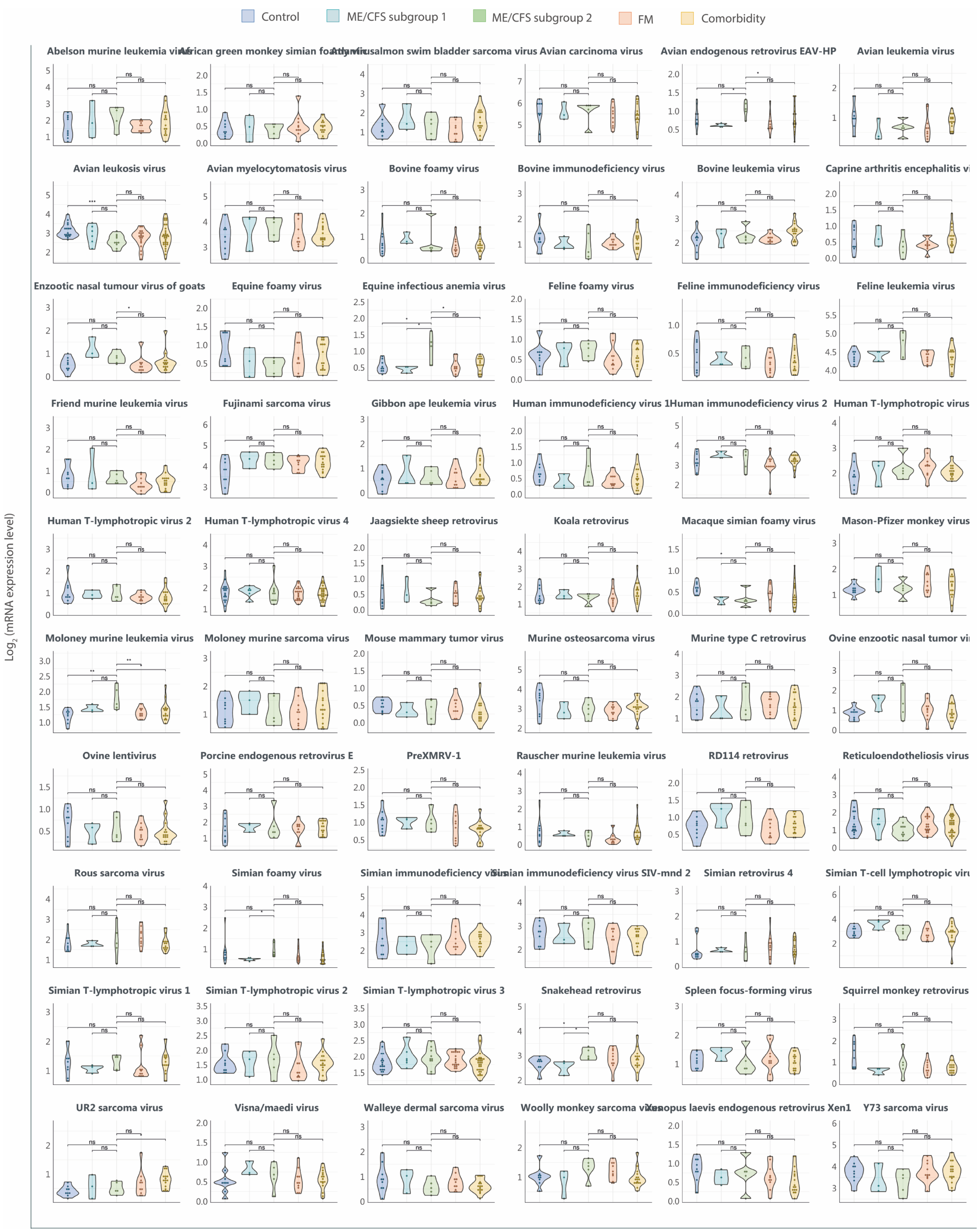

Figure S5. Detailed analysis of viruses belonging to the *Retroviridae* family. Plots summarize the distribution and expression levels of all probesets of the corresponding virus in each study group. Statistical tests: unpaired two-sample Wilcoxon Test with Benjamin-Hochberg p-value correction. (*p<0.05, **p<0.01, ns: non-significant).

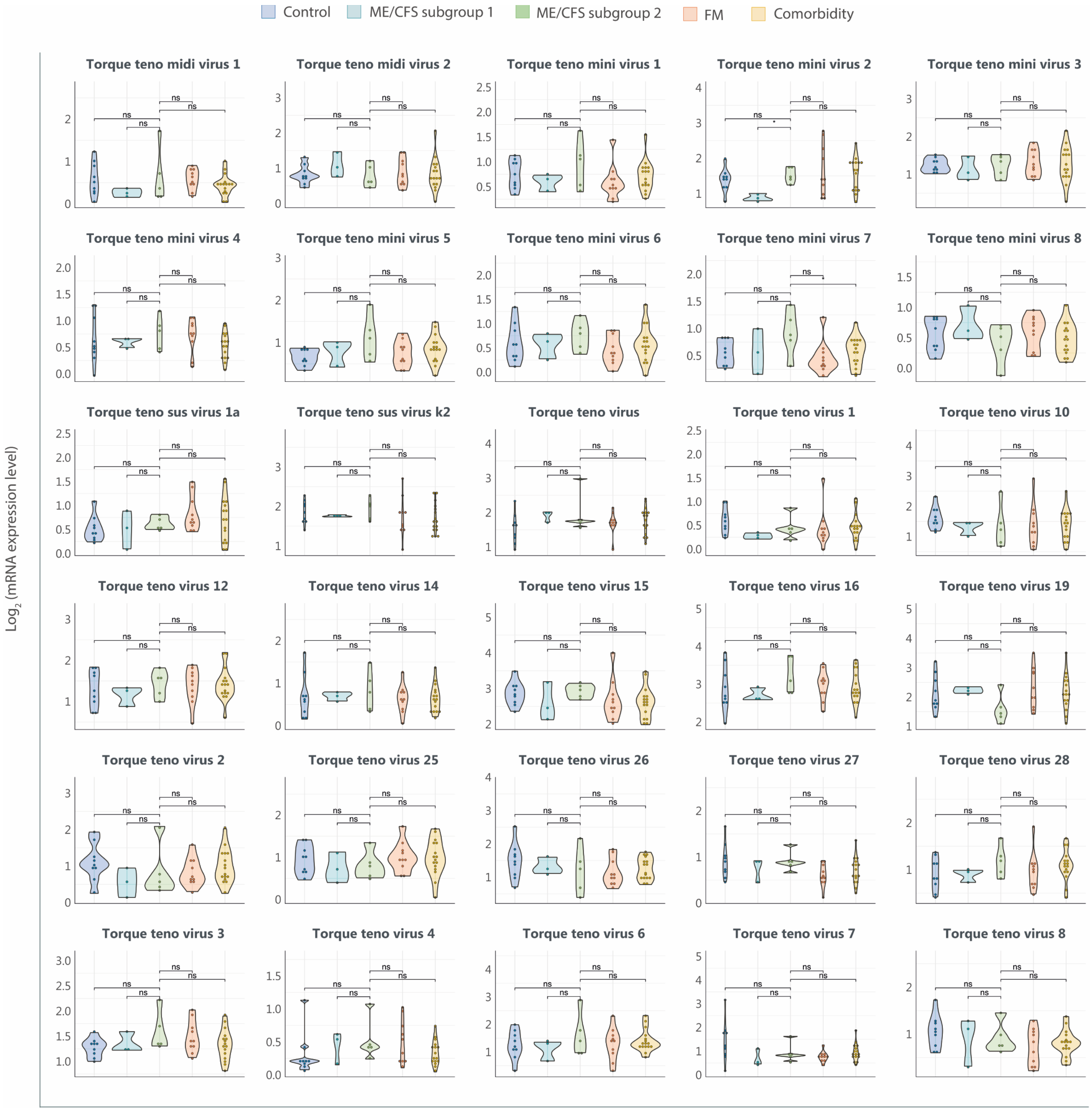

Figure S6. Detailed analysis of viruses belonging to the *Anelloviridae* family, except for TTMV9. Plots summarize the distribution and expression levels of all probesets of the corresponding virus in each study group. Statistical tests: unpaired two-sample Wilcoxon Test with Benjamin-Hochberg p-value correction. (*p<0.05, **p<0.01, ns: non-significant).
